# Flexural vibration systems with gyroscopic spinners

**DOI:** 10.1098/rsta.2019.0154

**Published:** 2019-09-02

**Authors:** G. Carta, M. J. Nieves, I. S. Jones, N. V. Movchan, A. B. Movchan

**Affiliations:** 1 Mechanical Engineering and Materials Research Centre, Liverpool John Moores University, Liverpool L3 3AF, UK; 2 School of Computing and Mathematics, Keele University, The Covert, Keele ST5 5BG, UK; 3 Department of Mechanical, Chemical and Material Engineering, University of Cagliari, Piazza d'Armi, 09123, Cagliari, Italy; 4 Department of Mathematical Sciences, University of Liverpool, Liverpool L69 7ZL, UK

**Keywords:** flexural waves, dispersion, chirality, gyroscopic spinners, gyrobeam

## Abstract

In this paper, we study the spectral properties of a finite system of flexural elements connected by gyroscopic spinners. We determine how the eigenfrequencies and eigenmodes of the system depend on the gyricity of the spinners. In addition, we present a transient numerical simulation that shows how a gyroscopic spinner attached to the end of a hinged beam can be used as a ‘stabilizer’, reducing the displacements of the beam. We also discuss the dispersive properties of an infinite periodic system of beams with gyroscopic spinners at the junctions. In particular, we investigate how the band-gaps of the structure can be tuned by varying the gyricity of the spinners.

This article is part of the theme issue ‘Modelling of dynamic phenomena and localization in structured media (part 1)’.

## 1. Introduction

When gyroscopic spinners are connected to elastic structured solids, they may alter the dynamic properties of these solids. In particular, when attached to the nodes of an elastic lattice, gyroscopic spinners change the dispersive and filtering properties of the system, in addition to the polarization of waves [1,2]. Gyroscopic spinners have also been used to create highly localized waveforms in an elastic lattice without the need of an interface [3] and to generate unidirectional edge and interfacial waves in a discrete medium [4–7]. Recently, a linearized formulation has been proposed to replace the spinner connected to the end of an elastic beam with effective boundary conditions, both when the beam end is hinged [8] and when it is free [9]. In [8,9], it was shown that the eigenfrequencies of an elastic beam can be tuned by changing the *gyricity* of the spinner, and that flexural waves are coupled with rotational motion.

Elastic beams with gyricity are referred to as *gyrobeams* in the literature. The theoretical model of a gyrobeam was initially presented in [10] and then further developed in [11–14]. Micropolar gyroelastic continua employing asymmetric Cosserat theory were discussed in [15,16]. This recent interest in gyrobeams is due to the potential applications of these special elements. For instance, they can be used to control the motion of spacecraft [17] and to reduce the vibrations of a structure subjected to seismic waves [18]. Despite being a theoretical structural element, a gyrobeam can be realized in practice as a periodic array of beams connected by gyroscopic spinners, as discussed in [8,9].

A system containing gyroscopic spinners possesses a property known as *chirality*. According to the original definition given in [19], an object is chiral if it cannot be superimposed onto its mirror image. Chirality has been employed in different types of lattices [20–22], auxetic media [23,24], homogenization analysis [25] and for the design of inertial resonators in a continuum [26]. In [27,28], it was shown how tilted resonators embedded in a triangular elastic lattice can change the dispersion surfaces of the medium and create localized waveforms. In this paper, we discuss dynamic phenomena associated with ‘active chirality’, produced by the mechanical action of gyroscopic spinners.

Microstructured and architected periodic media have attracted increasing attention in recent years, in parallel with novel concepts in structural mechanics [29], soft robotics [30,31] and locomotion [32]. In particular, new designs of lattice structures, exhibiting unusual dynamic properties, have been proposed. Dispersion degeneracies and localization in a grid of beams with rotational inertia, referred to as Rayleigh beams, were analysed in [33–35]. Pre-stress in two-dimensional arrays of axially and flexurally deformable beams was exploited in [36] to create negative refraction, total reflection and wave channelling. Discrete systems have been studied to predict crack propagation speed and explain crack tip instabilities [37–42]. Enhanced transmission, trapping and wave control with different types of resonators were investigated in [43–46]. In the present paper, we show how gyroscopic spinners can be implemented in a periodic system supporting flexural waves to stabilize a structure and to tune the filtering properties of the system.

The paper is organized as follows. In §2, we consider a finite array of flexural elements with gyroscopic spinners at the junctions. We determine how the eigenfrequencies and eigenfunctions of this system change with the properties of the spinners. In addition, we demonstrate with an illustrative numerical simulation that the motion of a beam hinged at one end can be ‘stabilized’ by attaching a gyroscopic spinner at the other end. In §3, we study an infinite periodic system made up of beams connected by gyroscopic spinners. We determine the dispersion curves for this structure and show how the pass- and stop-bands are modified by changing the gyricity of the spinners. In §4, we provide some concluding remarks.

## 2. Spectral problem for a finite system of beams connected by gyroscopic spinners

**Figure 1.**
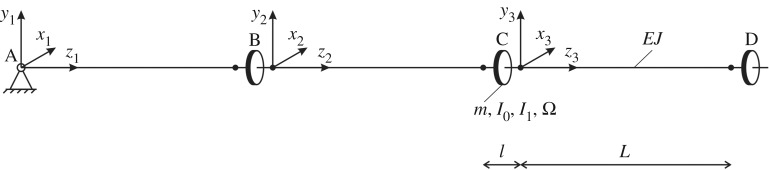
Finite system of beams attached to gyroscopic spinners and with a hinge at one end.

Here, we study the finite system shown in figure 1, consisting of three beams connected by gyroscopic spinners (at the junction points B and C). The system is hinged at one end (point A) and is connected to a gyroscopic spinner at the other end (point D). The lengths of the beams and the spinners are *L* and *l*, respectively, with *l*≪*L*. The flexural stiffness of the beams is *EJ* with respect to both the *x*_*j*_- and *y*_*j*_-axes (*j* = 1, 2, 3). Each spinner is a solid of revolution having mass *m*, moment of inertia *I*_1_ about its axis of revolution and moment of inertia *I*_0_ about the two transverse axes with origins at the spinner's base. Another important characteristic of the spinners is the *gyricity* $\Omega =\dot{\psi }+\dot{\phi }$, which is the sum of the spinner's spin rate $\dot{\psi }$ and precession rate $\dot{\phi }$ [8,9]. In [8,9], it has been shown that the gyricity is constant under the assumption of small nutation angle. Here, the beams are assumed to be massless, hence the inertia of the system is periodically concentrated at the junctions, where the gyroscopic spinners are placed.

Here, we solve formally an eigenvalue problem for the elastic gyroscopic system. In a practical way, this gives the basis for further transient analysis of solutions to Cauchy problems. For the case of massless beams, such solutions are explicit [9].

If the gyricity *Ω* = 0, the gyroscopic spinners behave like non-spinning and non-precessing rigid bodies with translational and rotational inertia. In this case, the first eigenfrequency of the system is expected to be zero. In the following, we will show that the first eigenfrequency of the system becomes positive when the gyroscopic effect is activated, namely when *Ω*≠0.

### (a) Eigenfrequencies as functions of the gyricity *Ω*

The governing equations for the massless beams are given by

2.1 $$u_{j}^{\prime \prime \prime \prime }\left(z_{j}\right)=0\;\mathrm{and}\;v_{j}^{\prime \prime \prime \prime }\left(z_{j}\right)=0,\;j=1,2,3,$$

where *u*_*j*_(*z*_*j*_) and *v*_*j*_(*z*_*j*_) are the displacement components along the local *x*_*j*_- and *y*_*j*_-directions, respectively, and the derivatives are taken with respect to the local coordinate *z*_*j*_ of the *j*th beam. The solutions of (2.1) are cubic functions of *z*_*j*_ and can be written as

2.2 $$\begin{array}{rl} & u_{j}\left(z_{j}\right)=A_{1}^{\left(j\right)}z_{j}^{3}+A_{2}^{\left(j\right)}z_{j}^{2}+A_{3}^{\left(j\right)}z_{j}+A_{4}^{\left(j\right)}\\ \;\mathrm{and}\; & v_{j}\left(z_{j}\right)=A_{5}^{\left(j\right)}z_{j}^{3}+A_{6}^{\left(j\right)}z_{j}^{2}+A_{7}^{\left(j\right)}z_{j}+A_{8}^{\left(j\right)},\;j=1,2,3, \end{array}\}$$

where the coefficients *A*^(*j*)^_*k*_ (*k* = 1, …, 8) are determined from the boundary and junction conditions.

At the hinge A, we impose zero transverse displacements and zero bending moments, namely

2.3 $$u_{1}(0)=v_{1}(0)=u_{1}^{\prime \prime }(0)=v_{1}^{\prime \prime }(0)=0.$$

At the junctions B and C, we replace the gyroscopic spinners with effective junction conditions, according to the analytical formulation developed in [8,9]. These are

2.4 $$u_{i}(L)=u_{i+1}(0),\;v_{i}(L)=v_{i+1}(0),\;u_{i}^{\prime }(L)=u_{i+1}^{\prime }(0),\;v_{i}^{\prime }(L)=v_{i+1}^{\prime }(0),\;i=1,2$$

and

2.5 $$\begin{array}{rl} & EJu_{i}^{\prime \prime }(L)=EJu_{i+1}^{\prime \prime }(0)+I_{0}\omega^{2}u_{i+1}^{\prime }(0)-i\omega I_{1}\Omega v_{i+1}^{\prime }(0),\\ & EJv_{i}^{\prime \prime }(L)=EJv_{i+1}^{\prime \prime }(0)+I_{0}\omega^{2}v_{i+1}^{\prime }(0)+i\omega I_{1}\Omega u_{i+1}^{\prime }(0),\\ & EJu_{i}^{\prime \prime \prime }(L)=EJu_{i+1}^{\prime \prime \prime }(0)-m\omega^{2}u_{i+1}(0)\\ \;\mathrm{and}\; & EJv_{i}^{\prime \prime \prime }(L)=EJv_{i+1}^{\prime \prime \prime }(0)-m\omega^{2}v_{i+1}(0),\;i=1,2. \end{array}\}$$

In the equations above, *ω* is the radian frequency of the system. At the end D, the following boundary conditions are set [8,9]:

2.6 $$\begin{array}{rl} & EJu_{3}^{\prime \prime }(L)=I_{0}\omega^{2}u_{3}^{\prime }(L)-i\omega I_{1}\Omega v_{3}^{\prime }(L),\;EJv_{3}^{\prime \prime }(L)=I_{0}\omega^{2}v_{3}^{\prime }(L)+i\omega I_{1}\Omega u_{3}^{\prime }(L)\\ \;\mathrm{and}\; & EJu_{3}^{\prime \prime \prime }(L)=-m\omega^{2}u_{3}(L),\;EJv_{3}^{\prime \prime \prime }(L)=-m\omega^{2}v_{3}(L). \end{array}\}$$

We note that the equations of angular momentum balance are coupled by the gyroscopic effect.

**Figure 2.**
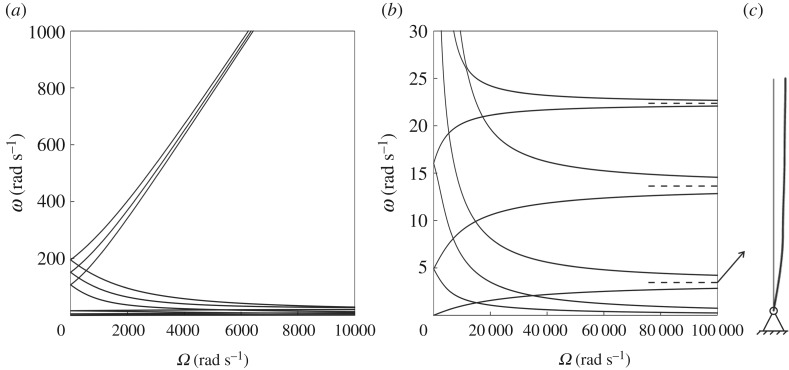
(*a*) Eigenfrequencies of the system in figure 1 versus the gyricity of the spinners; (*b*) a magnification of the results presented in (*a*) for 0≤*ω*≤30 rad s^−1^; (*c*) mode shape of the structure in the limit when *Ω* → ∞ for *ω* = 3.45 rad s^−1^.

Looking for non-trivial solutions of the homogeneous system (2.3)–(2.6), consisting of 24 equations in 24 unknown coefficients, we find the eigenfrequencies *ω* of the system. These eigenfrequencies are shown as functions of the gyricity *Ω* in figure 2*a*,*b*. In these illustrative numerical calculations, we have considered steel beams (*E* = 210 GPa) with length *L* = 6 m and square cross-section of side length 0.1 m. For the purpose of these calculations, the spinners are considered as steel cylinders (density *ρ* = 7850 kg m^−3^) of height *l* = *L*/10 and radius *r* = 2*l*.

When the spinners do not spin, i.e. *Ω* = 0, the structure exhibits five positive double eigenfrequencies, which are grouped in two distinct clusters. The analytical values of these double eigenfrequencies agree very well with those obtained from a finite-element model built in *Comsol Multiphysics*, representing a system of three beams with masses at their junctions having translational inertia *m* and rotational inertia *I*_0_. When *Ω* > 0, the double eigenfrequencies split into two distinct values and a null eigenfrequency becomes positive and increases with *Ω*. This implies that the spinners can act as ‘stabilizers’, preventing the structure from collapsing at low frequencies. The stabilizing effect of the gyroscopic spinners will be discussed in more detail in §2b.

In the limit when *Ω* → ∞, two curves in figure 2*a*,*b* tend to zero, three of them tend to infinity, and the remaining six converge to three finite double eigenfrequencies, indicated by dashed lines in figure 2*b*. These three limit values coincide with the eigenfrequencies of a system of three beams, with a hinge at point A and point masses assigned with zero rotation at the locations B, C and D (refer to figure 1). This limit case has been modelled in *Comsol Multiphysics* and an excellent agreement has been achieved. In figure 2*c*, we present the eigenmode in the limit when *Ω* → ∞ corresponding to the lowest of the three double eigenfrequencies, which shows that the structure maintains stability even if it is hinged at the bottom end.

The eigenmodes of the system for *Ω* = 200 rad s^−1^ are illustrated in the electronic supplementary material, Videos S1a–k. They correspond to the following eigenfrequencies: *ω*^(a)^ = 0.024 rad s^−1^, *ω*^(b)^ = 4.786 rad s^−1^, *ω*^(c)^ = 5.010 rad s^−1^, *ω*^(d)^ = 15.821 rad s^−1^, *ω*^(e)^ = 16.237 rad s^−1^, *ω*^(f)^ = 93.098 rad s^−1^, *ω*^(g)^ = 123.545 rad s^−1^, *ω*^(h)^ = 136.939 rad s^−1^, *ω*^(i)^ = 167.462 rad s^−1^, *ω*^(j)^ = 180.267 rad s^−1^ and *ω*^(k)^ = 210.942 rad s^−1^. The direction of rotation of the beam, clockwise or counter-clockwise, depends on the frequency. We also note that the positions of the stationary and inflection points along the beam change with frequency.

### (b) Stabilizing effect of gyroscopic spinners

**Figure 3.**
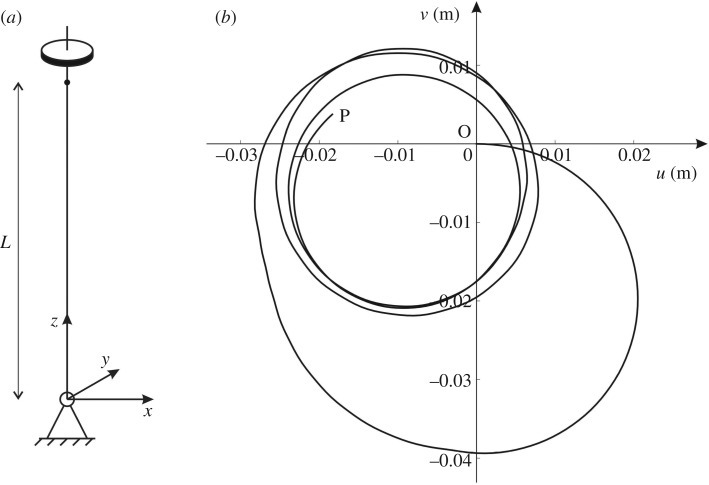
(*a*) Single beam hinged at *z* = 0 and with a gyroscopic spinner at *z* = *L*; (*b*) trajectory O−P of the tip of the beam in (*a*), subjected to an initial velocity in the *x*-direction.

Here, we show how a gyroscopic spinner can be used to ‘stabilize’ a single elastic beam. The beam is assumed to be hinged at *z* = 0 and attached to a gyroscopic spinner at *z* = *L* (figure 3*a*). The properties of the beam and the gyroscopic spinner are the same as those considered in §2a. In our model, gravity is neglected.

We perform a transient analysis of this system in *Comsol Multiphysics*, where the beam is modelled as a mono-dimensional element using Euler–Bernoulli theory and the spinner is replaced by the effective boundary conditions (2.6). We determine the transient response of the system for the spinner that has an initial velocity of 0.01 m s^−1^ in the *x*-direction. In the simulation, the gyricity is *Ω* = 1000 rad s^−1^ and the time step is 0.001 s. The trajectory of the beam end at *z* = *L* in the time interval [0, 50] s is shown in figure 3*b*. The initial position is the origin O of the *xy*-plane and the position at 50 s is denoted by P.

When the gyricity is non-zero, the tip of the beam maintains a trajectory in the vicinity of its initial position, as illustrated in figure 3*b*. When *Ω* = 0, the beam tip moves in the *xz*-plane undergoing a larger displacement than that for *Ω*≠0. We note that the larger the gyricity of the spinner, the smaller the amplitudes of the oscillations at the beam tip. Therefore, gyroscopic spinners can be used as stabilizers, reducing the vibrations of a structure. The motion of the system is further illustrated in the electronic supplementary material, Video S2.

### (c) Asymptotic approximation of the lowest eigenfrequency of the gyroscopic system

The lowest eigenfrequency of the gyroscopic system is extremely important for practical applications. In this section, we show how it depends on the geometrical and material parameters of the system.

For small values of the gyricity, the lowest eigenfrequency of the system in figure 1 is a linear function of *Ω*. The Taylor expansion of the dispersion relation around *Ω* = 0 leads to

2.7 $$\omega ∼\frac{3I_{1}}{3I_{0}+14mL^{2}}\Omega \;\mathrm{when}\;\left|\Omega \right|\ll 1.$$

For a more generic system of *N* beams and spinners, where the spinners can have different masses *m*^(*k*)^, different moments of inertia *I*^(*k*)^_0_, *I*^(*k*)^_1_ and different gyricities *Ω*^(*k*)^ throughout the structure, the asymptotic approximation of the lowest eigenfrequency has the form

2.8 $$\omega ∼\frac{1}{N}\frac{\sum_{k=1}^{N}I_{1}^{\left(k\right)}}{\sum_{k=1}^{N}I_{0}^{\left(k\right)}+L^{2}\sum_{k=1}^{N}k^{2}m^{\left(k\right)}}\sum_{k=1}^{N}\Omega^{\left(k\right)}\;\mathrm{when}\;\left|\Omega^{\left(k\right)}\right|\ll 1\;(k=1,\ldots ,N).$$

It is interesting to note that the lowest eigenfrequency can be zero even if the spinners have non-zero gyricities, but the sum of gyricities is zero. Formula (2.8) can be used in practice to choose the parameters of the system in order to make the structure stiffer or softer, depending on the applications.

## 3. Dispersion properties of a periodic system of beams connected by gyroscopic spinners

**Figure 4.**
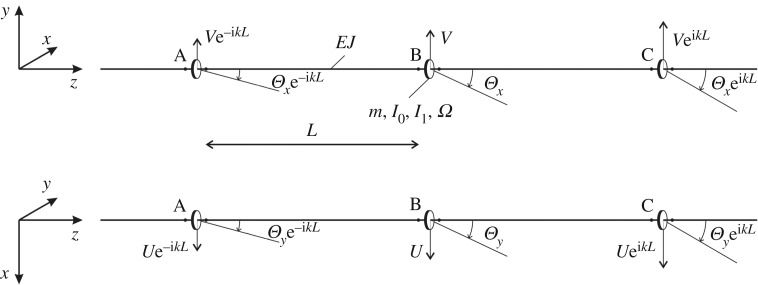
Periodic system made of elastic beams connected by gyroscopic spinners. The positive directions of the displacements and rotations of the structure in the time-harmonic regime are shown in both the *yz*- and *xz*-planes.

In this section, we investigate how Floquet–Bloch waves propagate in an infinite periodic structure consisting of elastic beams with gyroscopic spinners at the junctions. This structure is shown in figure 4, where the parameters characterizing the beams and the spinners are also indicated.

### (a) Equations of motion and dispersion relation

As in §2, we assume that the elastic beams are massless. Accordingly, at the *n*th junction the equations of motion of the spinner in the transient regime are given by [9]

3.1 $$\begin{array}{rl} & \frac{6EJ}{L^{3}}\left[2\left(u^{\left(n+1\right)}-2u^{\left(n\right)}+u^{\left(n-1\right)}\right)-\left(\theta_{y}^{\left(n+1\right)}-\theta_{y}^{\left(n-1\right)}\right)L\right]=m{\ddot{u}}^{\left(n\right)}\\ & \frac{6EJ}{L^{3}}\left[2\left(v^{\left(n+1\right)}-2v^{\left(n\right)}+v^{\left(n-1\right)}\right)-\left(\theta_{x}^{\left(n+1\right)}-\theta_{x}^{\left(n-1\right)}\right)L\right]=m{\ddot{v}}^{\left(n\right)},\\ & \frac{2EJ}{L^{2}}\left[3\left(v^{\left(n+1\right)}-v^{\left(n-1\right)}\right)-\left(\theta_{x}^{\left(n+1\right)}+4\theta_{x}^{\left(n\right)}+\theta_{x}^{\left(n-1\right)}\right)L\right]=I_{0}{\ddot{\theta }}_{x}^{\left(n\right)}-I_{1}\Omega {\dot{\theta }}_{y}^{\left(n\right)}\\ \;\mathrm{and}\; & \frac{2EJ}{L^{2}}\left[3\left(u^{\left(n+1\right)}-u^{\left(n-1\right)}\right)-\left(\theta_{y}^{\left(n+1\right)}+4\theta_{y}^{\left(n\right)}+\theta_{y}^{\left(n-1\right)}\right)L\right]=I_{0}{\ddot{\theta }}_{y}^{\left(n\right)}+I_{1}\Omega {\dot{\theta }}_{x}^{\left(n\right)}, \end{array}\}$$

where *u*^(*n*)^ = *u*^(*n*)^(*t*), *v*^(*n*)^ = *v*^(*n*)^(*t*) and *θ*^(*n*)^_*x*_ = *θ*^(*n*)^_*x*_(*t*), *θ*^(*n*)^_*y*_ = *θ*^(*n*)^_*y*_(*t*) are the displacements and rotations, respectively, at the *n*th junction in the transient regime. The dots denote derivatives with respect to time *t*.

We look for solutions of the form

3.2 $$\begin{array}{rl} & u^{\left(n\right)}(t)=Ue^{i\left(kLn-\omega t\right)},\;v^{\left(n\right)}(t)=Ve^{i\left(kLn-\omega t\right)}\\ \;\mathrm{and}\; & \theta_{x}^{\left(n\right)}(t)=\Theta_{x}e^{i\left(kLn-\omega t\right)}\;,\;\theta_{y}^{\left(n\right)}(t)=\Theta_{y}e^{i\left(kLn-\omega t\right)}, \end{array}\}$$

where *k* is the wavenumber and *U*, *V* , *Θ*_*x*_, *Θ*_*y*_ represent the amplitudes of displacements and rotations (see also figure 4). Using (3.2), system (3.1) can also be written in the following matrix form:

3.3 $$AU=\left(\begin{array}{cc} A_{11} & -A_{12}\\ A_{12} & A_{22} \end{array}\right)\left(\begin{array}{c} U\\ V\\ \Theta_{x}\\ \Theta_{y} \end{array}\right)=0,$$

where

3.4 $$\begin{array}{rl} & A_{11}=\left(\begin{array}{cc} m\omega^{2}-\frac{24EJ}{L^{3}}\left[1-\cos(kL)\right] & 0\\ 0 & m\omega^{2}-\frac{24EJ}{L^{3}}\left[1-\cos(kL)\right] \end{array}\right),\\ & A_{12}=\left(\begin{array}{cc} 0 & i\frac{12EJ}{L^{2}}\sin(kL)\\ i\frac{12EJ}{L^{2}}\sin(kL) & 0 \end{array}\right)\\ \;\mathrm{and}\; & A_{22}=\left(\begin{array}{cc} I_{0}\omega^{2}-\frac{4EJ}{L}\left[2+\cos(kL)\right] & iI_{1}\Omega \omega \\ -iI_{1}\Omega \omega & I_{0}\omega^{2}-\frac{4EJ}{L}\left[2+\cos(kL)\right] \end{array}\right). \end{array}\}$$

We note that the matrix ***A*** in (3.3) is Hermitian.

Non-trivial solutions of (3.3) are obtained by setting *det*(***A***) = 0, which yields

3.5 $$\begin{array}{rl} & {\left\{\left[m\omega^{2}L^{3}-24EJ(1-\cos(kL))\right]\left[I_{0}\omega^{2}L-4EJ(2+\cos(kL))\right]-144{\left(EJ\right)}^{2}\sin^{2}(kL)\right\}}^{2}\\ & \;-\Omega^{2}{\left\{I_{1}\omega L\left[m\omega^{2}L^{3}-24EJ(1-\cos(kL))\right]\right\}}^{2}=0. \end{array}$$

The equation above represents the dispersion relation of the system, which gives the eigenfrequencies *ω* of the structure as functions of the wavenumber *k*. When the spinners do not spin (*Ω* = 0), the dispersion relation has double roots. This is due to the fact that the cross-sections of the beams have been assumed to possess the same second moments of area about the *x*- and *y*-axes.

### (b) Dispersion curves

**Figure 5.**
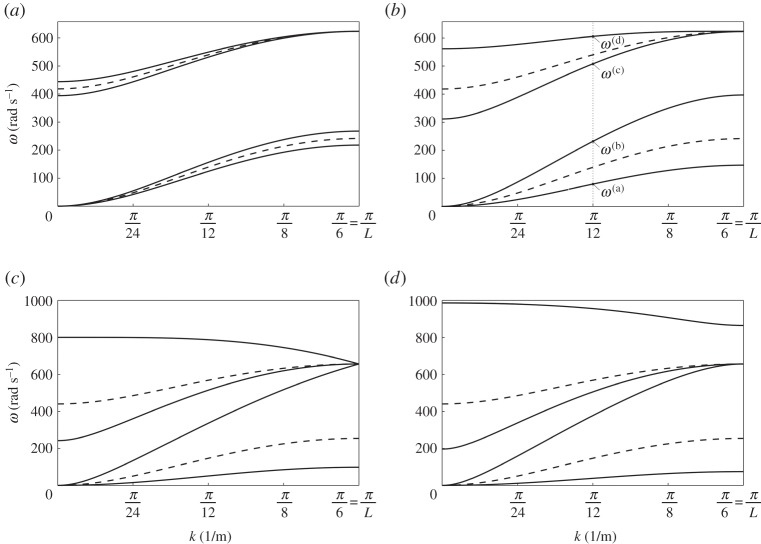
Dispersion curves of the periodic structure in figure 4 when (*a*) *Ω* = 100 rad s^−1^, (*b*) *Ω* = 500 rad s^−1^, (*c*) *Ω* = 1060.14 rad s^−1^ = Ω* and (*d*) *Ω* = 1500 rad s^−1^. The length of the periodic cell is *L* = 6 m. The dashed lines in (*a*)–(*d*) represent the case *Ω* = 0. We note that the scales of the vertical axes in (*a*) and (*b*) are different from those in (*c*) and (*d*).

We assume that the beams have Young's modulus *E* = 210 GPa, length *L* = 6 m and square cross-section of side length 0.1 m, as in the example of §2a. For the gyroscopic spinners, we take *m* = 1 kg, *I*_0_ = 20 kg m^2^ and *I*_1_ = 10 kg m^2^. The solid lines in figure 5*a*–*d* represent the dispersion curves of the system for different values of the gyricity *Ω*, specified in the caption. In the same diagrams, the dashed lines are the (double) dispersion curves of the system when the spinners are rigid bodies with mass *m*, rotational inertia *I*_0_ and zero gyricity.

From figure 5*a*,*b*, it is apparent that the main effect of gyricity is the splitting of each dashed line (denoting a double dispersion curve for *Ω* = 0) into two curves. This is analogous to the observation for the eigenfrequencies of the finite system discussed in §2 and shown in figure 2. The solid and dashed curves have two common points, namely (*k*, *ω*) = (0, 0) and $\left(k,\omega )=(\pi /L,4\sqrt{3\;EJ/mL^{3}}\right)$.

Figure 5*c* illustrates the situation when three dispersion curves have a common point at $\left(k,\omega )=(\pi /L,4\sqrt{3\;EJ/mL^{3}}\right)$. This occurs when the gyricity *Ω* is equal to

3.6 $$\Omega^{*}=\frac{\sqrt{EJ}\left(12I_{0}-mL^{2}\right)}{\sqrt{3mL^{3}}I_{1}}.$$

With our choice of the values of the parameters, we find Ω* = 1060.14 rad s^−1^. We note that the group velocities for all dispersion curves at *k* = *π*/*L* are equal to zero.

When *Ω* > Ω*, the highest dispersion curve moves upwards and a new internal stop-band is generated. The dispersion curves for *Ω* = 1500 rad s^−1^ > Ω* are presented in figure 5*d*.

The results of figure 5*a*–*d* show that the positions and the widths of pass- and stop-bands of such a gyroscopic system can be controlled by changing the gyricity of the spinners. Consequently, this system can be very useful in practical applications based on filtering of elastic waves.

### (c) Vibrational modes of the periodic system

Given the values of *k* and *ω* that satisfy the dispersion equation (3.5), the eigenfunctions for the elementary cell of the periodic structure in figure 4 are determined as non-trivial solutions of the homogeneous system (3.3).

In any massless beam of the periodic system, the displacements in the *x*- and *y*-directions are given by

3.7 $$\begin{array}{r} U(z)=\frac{2\left(U^{\left(l\right)}-U^{\left(r\right)}\right)+\left(\Theta_{y}^{\left(l\right)}+\Theta_{y}^{\left(r\right)}\right)L}{L^{3}}z^{3}+\frac{3\left(U^{\left(r\right)}-U^{\left(l\right)}\right)-\left(2\Theta_{y}^{\left(l\right)}+\Theta_{y}^{\left(r\right)}\right)L}{L^{2}}z^{2}+\Theta_{y}^{\left(l\right)}z+U^{\left(l\right)} \end{array}$$

and

3.8 $$\begin{array}{r} V(z)=\frac{2\left(V^{\left(l\right)}-V^{\left(r\right)}\right)+\left(\Theta_{x}^{\left(l\right)}+\Theta_{x}^{\left(r\right)}\right)L}{L^{3}}z^{3}+\frac{3\left(V^{\left(r\right)}-V^{\left(l\right)}\right)-\left(2\Theta_{x}^{\left(l\right)}+\Theta_{x}^{\left(r\right)}\right)L}{L^{2}}z^{2}+\Theta_{x}^{\left(l\right)}z+V^{\left(l\right)}, \end{array}$$

respectively. In (3.7) and (3.8), 0≤*z*≤*L* and the superscript ‘(l)’ denotes the value calculated at the left end of the beam *z* = 0 and the superscript ‘(r)’ the value at the right end of the beam *z* = *L*.

**Figure 6.**
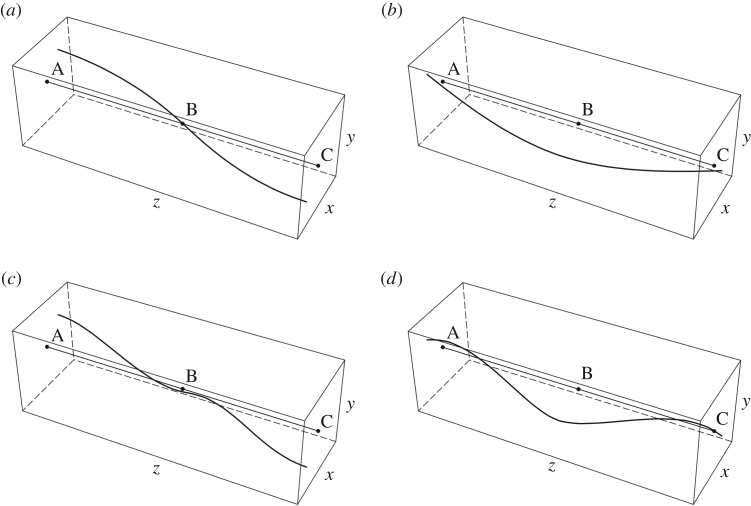
Vibrational modes of the periodic system in figure 4, calculated for *Ω* = 500 rad s^−1^, *k* = *π*/12 1/m and (*a*) *ω* = 79.565 rad s^−1^ = *ω*^(a)^, (*b*) *ω* = 231.757 rad s^−1^ = *ω*^(b)^, (*c*) *ω* = 507.826 rad s^−1^ = *ω*^(c)^, (*d*) *ω* = 605.634 rad s^−1^ = *ω*^(d)^.

The eigenfunctions of the periodic system for *Ω* = 500 rad s^−1^ and *k* = *π*/(2*L*) are presented in figure 6*a*–*d*. Only two spans of the periodic structure are shown in the figures. The corresponding eigenfrequencies are *ω*^(a)^ = 79.565 rad s^−1^, *ω*^(b)^ = 231.757 rad s^−1^, *ω*^(c)^ = 507.826 rad s^−1^ and *ω*^(d)^ = 605.634 rad s^−1^ (figure 5*b*). The locations of the stationary and inflection points along the *z*-axis vary with the eigenfrequency.

The eigenfunctions are also illustrated in the electronic supplementary material, Videos 3a–3d. Each point of the beam axis describes a circular trajectory. When *ω* = *ω*^(a)^ and *ω* = *ω*^(c)^, the rotation of each point around the *z*-axis is in the clockwise direction, while for *ω* = *ω*^(b)^ and *ω* = *ω*^(d)^, it is in the counter-clockwise direction.

## 4. Conclusion

We have shown that the spectral properties of a flexural system can be altered significantly by introducing the chirality action produced by gyroscopic spinners. This may be of utmost importance in many practical engineering applications, where resonant effects can lead to the collapse of a structure or a building.

Gyroscopic spinners can also be employed to stabilize a structural element. By means of a transient numerical computation, we have demonstrated that a beam, hinged at one end and subjected to an initial disturbance, can be ‘stabilized’ by attaching a gyroscopic spinner at its other end. Conversely, in the absence of the gyroscopic spinner, the same beam would undergo large displacements. The stabilizing effect of gyroscopic spinners can be exploited in the design and construction of structures subjected to dynamic loading.

A periodic flexural system with gyroscopic spinners can be very useful in the context of wave filtering. In particular, the gyricity of the spinners can be varied in order to change the widths and positions of the stop-bands, depending on the requirements.

## Supplementary Material

Video 1a

## Supplementary Material

Video 1b

## Supplementary Material

Video 1c

## Supplementary Material

Video 1d

## Supplementary Material

Video 1e

## Supplementary Material

Video 1f

## Supplementary Material

Video 1g

## Supplementary Material

Video 1h

## Supplementary Material

Video 1i

## Supplementary Material

Video 1j

## Supplementary Material

Video 1k

## Supplementary Material

Video 2

## Supplementary Material

Video 3a

## Supplementary Material

Video 3b

## Supplementary Material

Video 3c

## Supplementary Material

Video 3d

## Data accessibility

The paper contains no experimental data. Computations were performed using *Wolfram Mathematica* (version 10) and *Comsol Multiphysics* (v. 5.3).
