# Supplementary figures and images for: Flexural vibration systems with gyroscopic spinners
Source: Philos Trans A Math Phys Eng Sci. 2019 Sep 2;377(2156):20190154. doi: 10.1098/rsta.2019.0154 (PMC6732376; doi:10.1098/rsta.2019.0154)

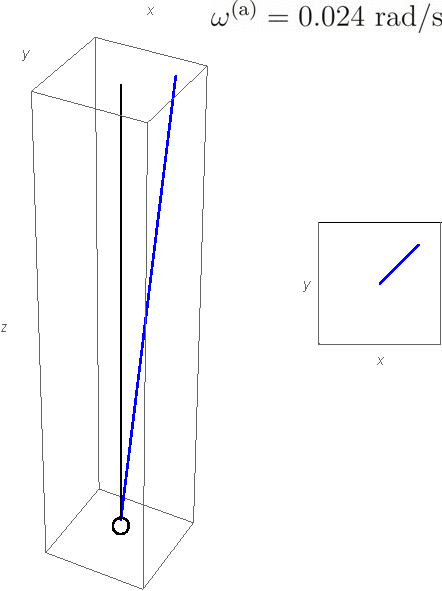

Supplement: Video 1a [file rsta20190154supp1.gif]

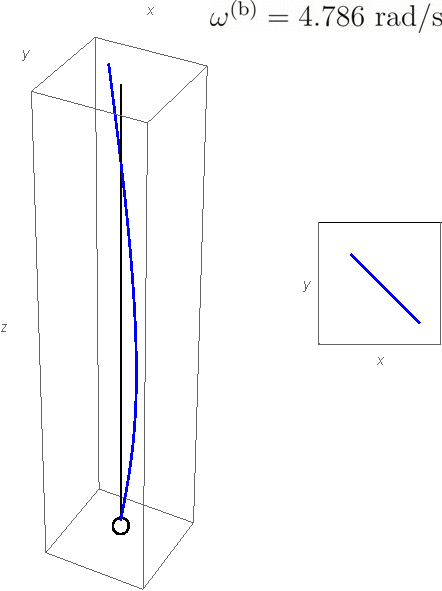

Supplement: Video 1b [file rsta20190154supp2.gif]

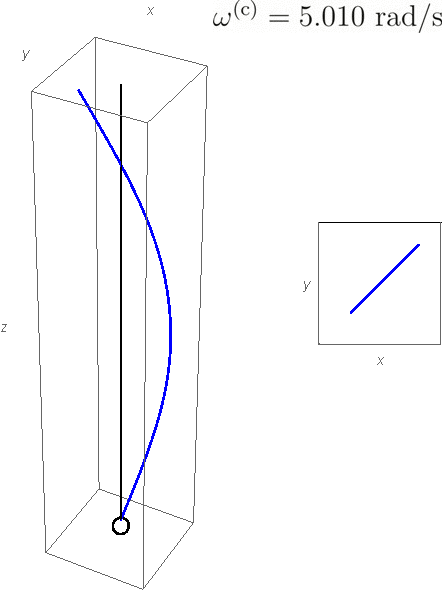

Supplement: Video 1c [file rsta20190154supp3.gif]

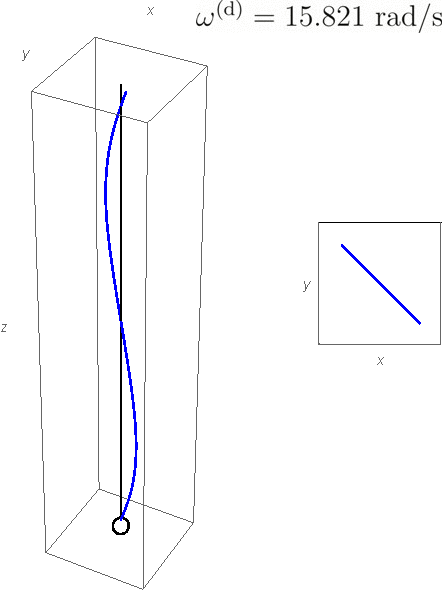

Supplement: Video 1d [file rsta20190154supp4.gif]

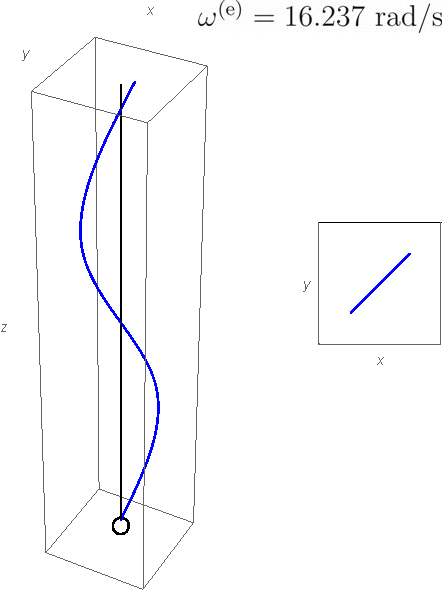

Supplement: Video 1e [file rsta20190154supp5.gif]

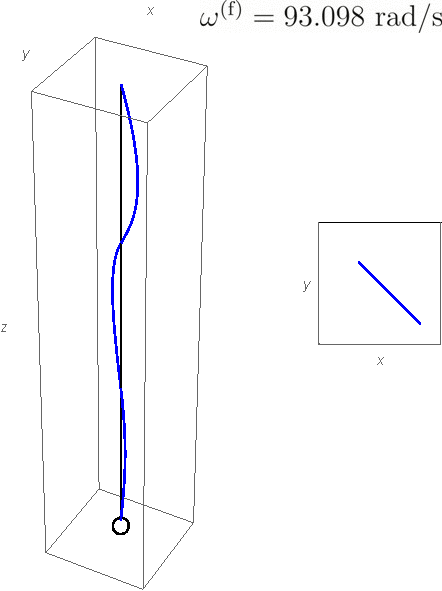

Supplement: Video 1f [file rsta20190154supp6.gif]

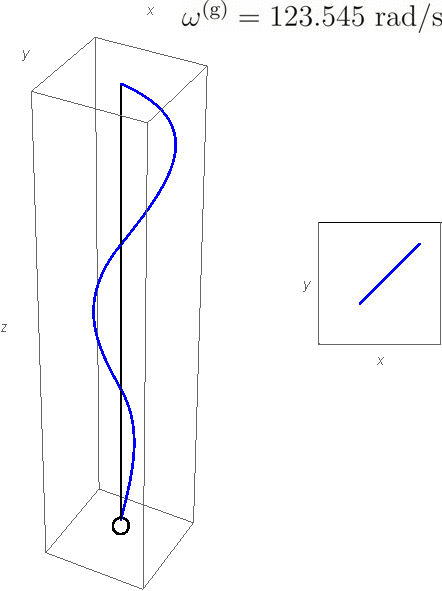

Supplement: Video 1g [file rsta20190154supp7.gif]

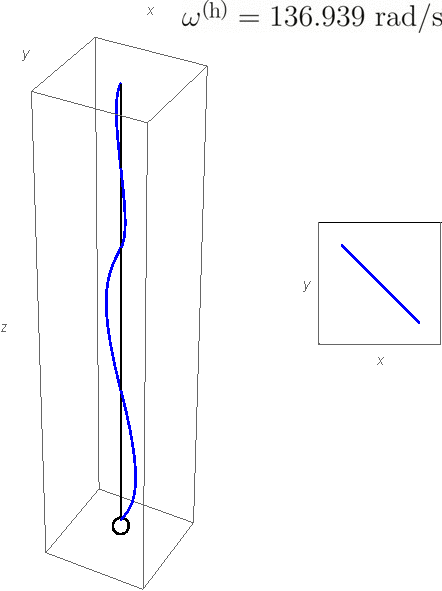

Supplement: Video 1h [file rsta20190154supp8.gif]

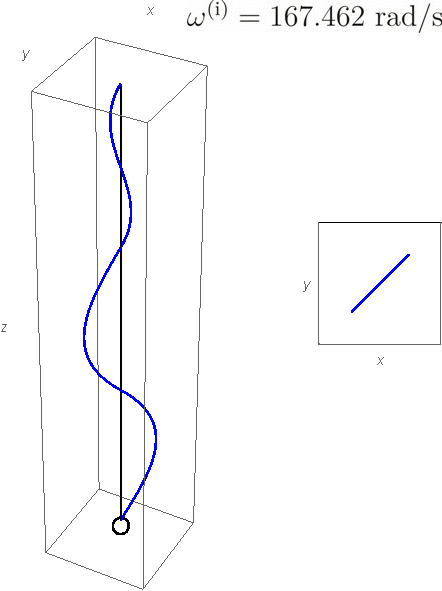

Supplement: Video 1i [file rsta20190154supp9.gif]

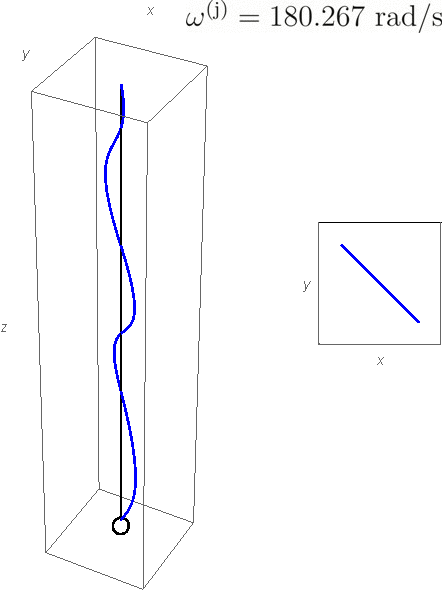

Supplement: Video 1j [file rsta20190154supp10.gif]

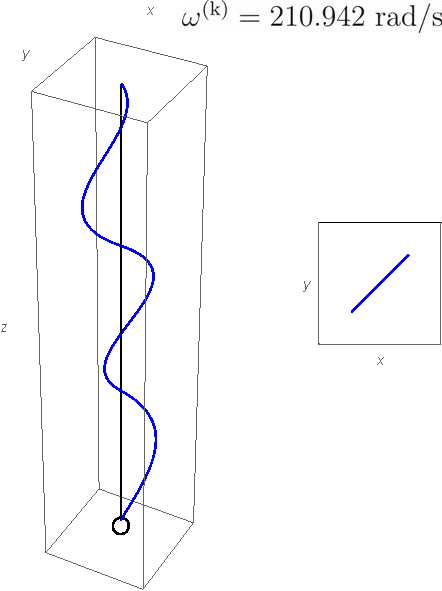

Supplement: Video 1k [file rsta20190154supp11.gif]

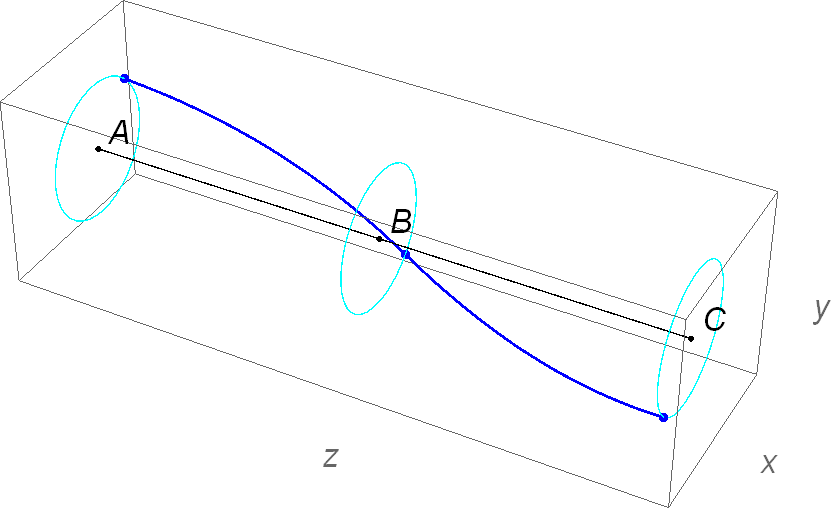

Supplement: Video 3a [file rsta20190154supp13.gif]

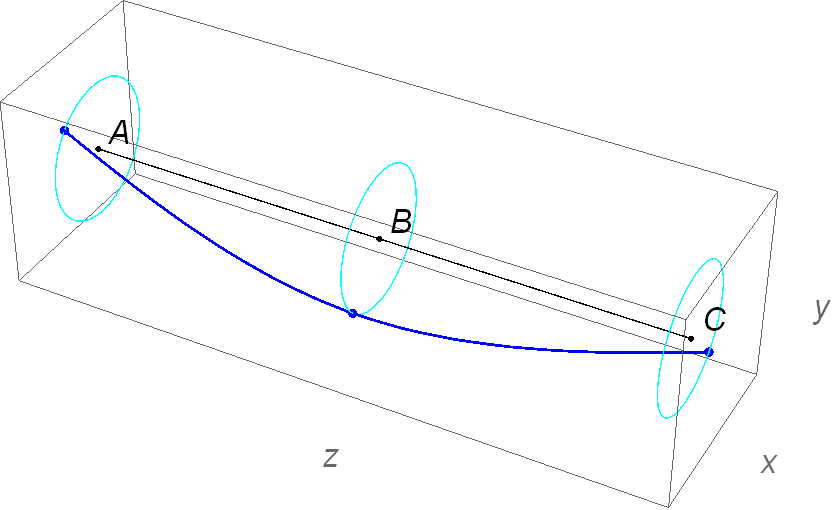

Supplement: Video 3b [file rsta20190154supp14.gif]

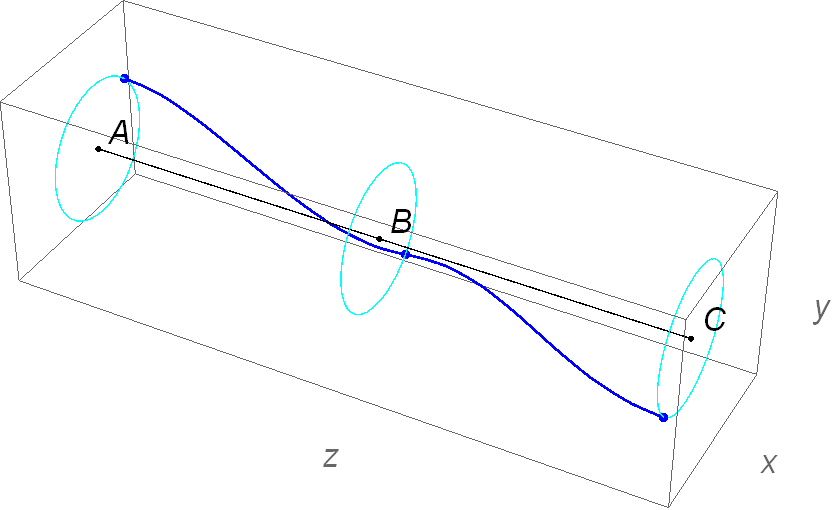

Supplement: Video 3c [file rsta20190154supp15.gif]

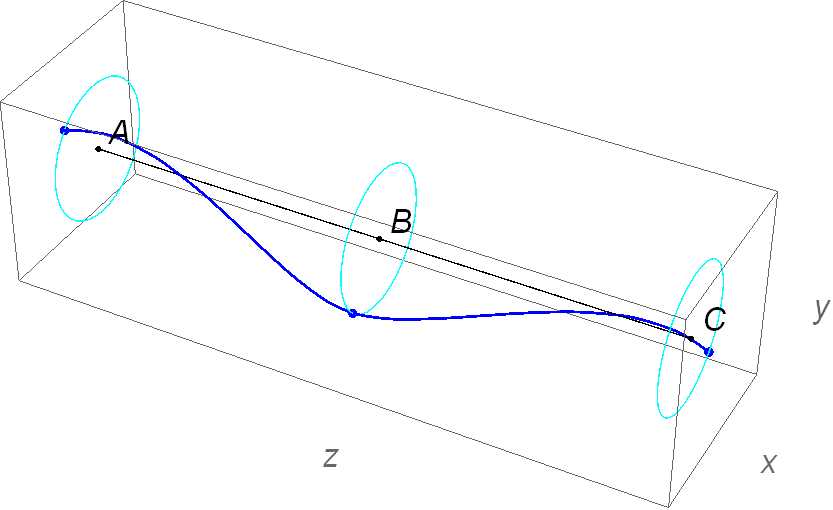

Supplement: Video 3d [file rsta20190154supp16.gif]
